# Immune Checkpoint Inhibitors and Bone Health: Mechanisms, Clinical Implications, and Emerging Perspectives on Bone Fragility

**DOI:** 10.1007/s11914-026-00952-7

**Published:** 2026-03-05

**Authors:** Muhammed Emin Bedir, Abdulhadi Cihangir Uguz, Ismail Beypinar, Mustafa Unal

**Affiliations:** 1 Department of Biophysics, Faculty of Medicine, Karamanoğlu Mehmetbey University, Karaman, 70200 Türkiye; 2 Department of Electric and Energy, Vocational School of Technical Sciences, Karamanoğlu Mehmetbey University, Karaman, 70200 Türkiye; 3 Department of Medical Physics, University of Wisconsin-Madison, Madison, WI 53707 USA; 4 Department of Oncology, Alanya Alaaddin Keykubat University, Alanya, Antalya, 07450 Türkiye; 5 The Center for Advanced Orthopedics Studies, Beth Israel Deaconess Medical Center, Boston, 02015 MA USA; 6 Harvard Medical School, Department of Orthopedic Surgery, Boston, 02015 MA USA; 7 Health Institutes of Türkiye (TÜSEB), Aziz Sancar Research Center, Ankara, 06270 Türkiye; 8 BIDMC Department of Orthopedic Surgery, Harvard Medical School, Boston, MA 02015 USA

**Keywords:** Immune Checkpoint Inhibitors, Bone Fragility, Bone Quality, Bone remodeling, Osteoimmunology

## Abstract

**Purpose of Review:**

Immune checkpoint inhibitors (ICIs) have revolutionized cancer treatment, but their effects on the skeletal system, independent of the influence of bone metastases, are not fully understood. This critical review specifically summarizes the current evidence on the systemic skeletal effects of ICIs in patients without bone metastases, focusing on changes in bone turnover markers (BTMs), bone mineral density (BMD), and fracture risk, thereby addressing a significant gap in the literature. It also explores potential mechanisms, such as immune-mediated disruption of bone remodeling and alterations in bone quality.

**Recent Findings:**

Preclinical in vivo models consistently report that blocking the programmed cell death protein 1/programmed death-ligand 1 (PD-1/PD-L1) or cytotoxic T-lymphocyte-associated antigen 4 (CTLA-4) pathways leads to bone loss and increased osteoclast activity. However, clinical findings are paradoxical; while one study using opportunistic quantitative computed tomography (QCT) reported preserved or even improved BMD in patients on ICI therapy, large cohort studies and pharmacovigilance analyses have revealed a consistently elevated risk of fragility fractures. Current assessment tools have limitations in capturing the true skeletal burden of ICI therapy.

**Summary:**

A significant discrepancy exists between preclinical data suggesting bone loss and clinical data showing both stable BMD and increased fracture risk. Addressing these knowledge gaps through prospective, high-resolution studies is critical for improving survivorship care. A clearer understanding is essential for developing strategies to prevent skeletal complications in the growing population of patients receiving immunotherapy.

## Introduction

Over the past decade, cancer therapy has undergone a profound transformation with the advent and widespread clinical integration of immune checkpoint inhibitors (ICIs). ICIs have revolutionized oncological treatment approaches by targeting inhibitory immune pathways, most notably the programmed cell death protein 1 (PD-1)/ programmed death-ligand 1 (PD-L1) axis and the cytotoxic T-lymphocyte-associated antigen 4 (CTLA-4) pathway. These agents function by “*releasing the brakes*” on cytotoxic T lymphocytes, thereby enabling a more potent and sustained antitumor immune response [1, 2]. Clinically approved ICIs include anti-CTLA-4 antibodies such as ipilimumab and tremelimumab; anti-PD-1 antibodies including nivolumab, pembrolizumab, and cemiplimab; and anti-PD-L1 antibodies such as atezolizumab, durvalumab, and avelumab [3]. Their application has led to significant and durable responses across a wide range of malignancies, including melanoma, non-small cell lung cancer, and renal cell carcinoma, with expanding indications in earlier stages of the disease and combination regimens [1, 2, 4]. Consequently, increasing numbers of patients are receiving these agents over extended durations, shifting the clinical focus toward long-term safety, survivorship, and the comprehensive management of immune-related adverse events (irAEs) [5].

Although irAEs affecting the skin, gastrointestinal tract, liver, and endocrine glands are well characterized [2, 6–8], the systemic skeletal effects of ICIs remain poorly understood and are often excluded from standard irAE profiles [9, 10]. As more patients benefit from prolonged survival, the integrity of the skeletal system becomes an increasingly important consideration in survivorship care. Bone fragility, leading to fractures, impaired mobility, and chronic pain, poses a substantial threat to quality of life and may even indirectly affect oncological outcomes by limiting physical activity [4]. Initial pharmacovigilance reports and retrospective analyses have raised concerns about increased fracture risk in ICIs-treated patients [9, 11, 12], but comprehensive clinical data on bone turnover markers (BTMs), bone mineral density (BMD), and fracture incidence remain sparse and often inconsistent [10, 13].

The skeletal system is not immunologically inert. Bone homeostasis is maintained through the coordinated activities of osteoclasts and osteoblasts, which are regulated within basic multicellular units (BMUs) under the control of osteocytes [14, 15]. This remodeling process is subject to complex immune modulation in the bone (i.e., osteoimmunology) [1, 16, 17]. T lymphocytes, in particular, are known to exert profound effects on bone metabolism through both direct cell-cell interactions and cytokine secretion. Among the most critical pathways is the receptor activator of nuclear factor kappa-B ligand (RANKL) axis, which is central to osteoclast differentiation and is expressed not only by stromal and bone cells, but also by activated T-cells [15]. Pro-inflammatory cytokines such as tumor necrosis factor-alpha (TNF-α), interleukin-1 (IL-1), interleukin-6 (IL-6), interleukin-17 (IL-17) are known to promote bone resorption [16]. The role of interferon-gamma (IFN-γ) is more complex; as it can exert both anti-resorptive and pro-resorptive effects depending on the specific inflammatory context [18, 19]. In contrast, regulatory cytokines such as IL-4 and those derived from Treg cells may inhibit osteoclastogenesis [20].

Importantly, checkpoint molecules targeted by ICIs are also involved in physiological bone remodeling. CTLA-4 interacts with CD80/CD86 on antigen-presenting cells, including osteoclast precursors, and has been shown to suppress osteoclast formation through direct effects on osteoclast precursors [21]. Similarly, PD-1 and its ligands (PD-L1, PD-L2) are expressed on various immune and non-immune cells within the bone microenvironment, including osteoblasts and potentially osteoclasts [9, 12, 22–24]. Experimental models have demonstrated that the deletion of these molecules leads to an osteoporotic bone phenotype, underscoring their relevance in skeletal homeostasis [22, 25]. However, it should be noted that the specific phenotype can be contradictory, with some early studies of PD-1 deficient mice reporting mild osteopetrosis (bone gain) [20]. A central tenet of osteoimmunology is that T-lymphocytes are potent regulators of bone metabolism [16]. The nature of this regulation, however, is highly dependent on the context and the specific inflammatory milieu, as demonstrated by the opposing effects of activated versus non-activated T-cells on osteoclastogenesis [26]. In well-established models of pathological bone loss, such as that induced by estrogen deficiency or in rheumatoid arthritis, the activation of T-cells is a key driver of increased bone resorption [15]. In these conditions, activated T-cells enhance osteoclastogenic signaling through the secretion of pro-inflammatory cytokines, most notably RANKL and TNF-α [16]. Given this mechanistic overlap, it is hypothesized that the systemic T-cell activation induced by ICIs may mimic these pathological inflammatory states, thereby disturbing the balance between bone formation and resorption independent of their antitumor action [1].

However, the current understanding of these systemic skeletal effects remains unclear. While inflammatory musculoskeletal complications, such as arthralgia and myositis, are increasingly recognized as irAEs, reports of changes in BMD, BTMs, and fracture risk point toward a distinct and direct interference with bone remodeling processes [27]. This raises a clinically relevant and timely question: do ICIs exert systemic effects on the bone microenvironment that compromise bone strength, microarchitecture, and mineralization, independent of metastatic disease?

To date, the literature on ICIs-induced bone effects is limited and heterogeneous, with discrepancies between preclinical findings, observational data, and real-world clinical outcomes. A recent review by Joseph et al. [28] has laid the groundwork for this emerging topic; however, the rapid evolution of dynamic research in this area necessitates an updated and more comprehensive synthesis. The aim of this critical review is to critically examine the systemic skeletal effects of ICIs (specifically anti-PD-1, anti-PD-L1, and anti-CTLA-4 agents), focusing on their impact on bone cell function, the bone microenvironment (BME), and clinically measurable outcomes such as BMD, BTMs, and fracture incidence. In doing so, we sought to clarify the mechanisms by which these agents may alter bone physiology, delineate their clinical implications for non-metastatic bone health, and identify key areas for future research that are essential for optimizing both oncological and musculoskeletal outcomes in cancer patients receiving ICIs therapy.

## Immune Checkpoint Inhibitors: Mechanisms and Immunomodulatory Effects

As introduced, ICIs reinvigorate cytotoxic T-lymphocytes (CTLs) by blocking the key inhibitory pathways that tumors exploit to evade immune detection and destruction [28, 29]. Rather than acting directly on tumor cells, ICIs reinvigorate cytotoxic T-lymphocytes (CTLs) by blocking the key inhibitory pathways that tumors exploit to evade immune detection and destruction [28, 29]. The two principal checkpoint pathways targeted in clinical oncology are the PD-1/PD-L1 and CTLA-4 axes [30, 31].

The PD-1 receptor (programmed death-1), expressed on activated T-cells, B cells, natural killer (NK) cells, and myeloid cells, serves as a brake on the immune response. Its ligands, PD-L1 and PD-L2, are expressed not only on tumor cells and antigen-presenting cells (APCs), but also on various non-hematopoietic tissues, including cells within the bone microenvironment [9, 12]. The engagement of PD-L1 or PD-L2 with PD-1 dampens T-cell receptor signaling, promotes T-cell exhaustion, and facilitates immune tolerance, allowing tumor cells to persist. Therapeutic antibodies such as nivolumab, pembrolizumab, and cemiplimab (anti-PD-1), and atezolizumab, durvalumab, and avelumab (anti-PD-L1), disrupt this interaction, thereby restoring T-cell effector function and promoting anti-tumor immunity [12].

In contrast, CTLA-4 (cytotoxic T-lymphocyte-associated protein 4) is predominantly expressed on regulatory T-cells (Tregs) and activated conventional T-cells. It competes with the costimulatory receptor CD28 for binding to the ligands CD80 (B7-1) and CD86 (B7-2) on APCs [32]. Engagement of CTLA-4 leads to inhibitory signaling, particularly during the initial priming of naive T-cells in lymphoid organs, thereby attenuating immune activation at an early stage. Therapeutic blockade of this interaction using antibodies, such as ipilimumab and tremelimumab, enhances T-cell proliferation and effector function while reducing Tregs-mediated suppression [12, 33].

The therapeutic reactivation of immune surveillance through checkpoint blockade facilitates not only the infiltration of tumors by effector T-cells but also the production of pro-inflammatory cytokines such as IFN-γ and TNF-α [1]. Although these effects are instrumental in promoting tumor regression, the systemic nature of immune activation also underlies the occurrence of irAEs, wherein the loss of self-tolerance results in immune-mediated damage to healthy tissues. While most clinical studies have focused on irAEs affecting the gastrointestinal, endocrine, and dermatological systems, increasing attention has been directed toward musculoskeletal and skeletal complications [2, 34].

The mechanistic distinctions between CTLA-4 and PD-1/PD-L1 inhibitors may have important implications for their skeletal effects. CTLA-4 inhibitors act predominantly at the level of secondary lymphoid organs, enhancing the priming of T-cells broadly, which may result in more generalized immune activation. PD-1/PD-L1 inhibitors, by contrast, exert their effects primarily in peripheral tissues and within the tumor microenvironment, including the BME, particularly where PD-L1 is expressed on local cells [35]. This spatial difference in action could underline varying skeletal toxicity profiles. It has been hypothesized that CTLA-4 inhibition, due to its upstream and systemic immune modulation, might induce earlier and more diffuse effects on bone remodeling by broadly enhancing T-cell activation [35]. In contrast, PD-1/PD-L1 blockade may exert more targeted effects within the bone tissue itself, where PD-1-expressing T-cells interact with PD-L1-expressing bone cells [35, 36].

This hypothesis is partially supported by the pharmacovigilance data. For instance, a large-scale analysis by [34] found that pembrolizumab was associated with the highest overall risk of musculoskeletal adverse events, while ipilimumab showed the lowest toxicity in musculoskeletal and connective tissue disorders. Although this might appear counterintuitive, given that CTLA-4 inhibitors are generally more immunostimulatory, it may reflect differences in PD-L1 expression in musculoskeletal tissues or variations in the T cell subpopulations affected by PD-1 versus CTLA-4 blockade. Furthermore, preclinical studies indicate that genetic or pharmacological disruption of PD-1 signaling directly enhances osteoclastogenesis and reduces bone mass [20, 22, 25], establishing a more direct link between PD-1 inhibition and bone loss. Although CTLA-4 blockade also leads to T-cell activation and enhanced RANKL production, direct comparative studies assessing their respective effects on osteoblast and osteoclast function, bone turnover, and microarchitecture remain limited. Such studies are crucial for evaluating whether specific ICIs classes confer differential skeletal risk and for developing mitigation strategies that are altered by the immunotherapy regimen being used.

As ICIs continue to expand in indications and patient populations, understanding the nuanced mechanisms by which each agent modulates immune responses within the skeletal system will be essential. This knowledge could eventually inform personalized treatment approaches that balance oncologic efficacy with musculoskeletal safety, particularly for long-term cancer survivors at a risk of osteoporosis or fragility fractures.

## The Bone–Immune Axis: Cellular Players and Key Signaling Pathways

The interaction between skeletal and immune systems (osteoimmunology) offers a critical framework for understanding how ICIs affect bone physiology [1, 28]. This bidirectional communication is essential not only for maintaining bone homeostasis, but also for elucidating the pathogenesis of bone disorders, including those potentially triggered by immunotherapies. Rather than being an inert structural scaffold, the BME represents an active immunological compartment, populated by a diverse array of cells that continuously interact. Given that ICIs exert systemic effects by modulating immune responses, it is logical to consider their engagement with the BME as intrinsic to their mechanism of action, rather than as collateral or unrelated effects.

The BME comprises multiple cell types that participate in complex signaling networks. Osteoclasts, derived from hematopoietic precursors of the monocyte/macrophage lineage, are specialized for bone resorption and are essential for maintaining calcium homeostasis and bone integrity Osteoblasts, which originate from mesenchymal stem cells, are responsible for bone matrix synthesis and mineralization, and also modulate osteoclast activity. Osteocytes, which are terminally differentiated osteoblasts embedded in a mineralized matrix, act as mechanosensory and orchestrate bone remodeling by regulating both osteoblast and osteoclast functions. They secrete key signaling molecules such as RANKL, osteoprotegerin (OPG), sclerostin, and Dickkopf-1 (DKK1), thereby influencing both bone resorption and formation [37].

In addition to bone cells, the BME contains an extensive repertoire of immune cells. The bone marrow, as the primary site of hematopoiesis, hosts T-lymphocytes, B-lymphocytes, dendritic cells, macrophages, and myeloid-derived suppressor cells (MDSCs), among others [28]. These immune cells not only develop in the marrow but also interact continuously with bone-resident cells, shaping the local and systemic bone-immune interface [1]. This interaction is bidirectional and occurs in several capacities; for instance, activated T-cells are a major source of the pro-resorptive cytokine RANKL, while B-cells can produce the anti-resorptive decoy receptor OPG, directly influencing bone cell activity [16]. Given that the primary effect of ICIs is the systemic activation and proliferation of T-cells, these agents inevitably influence this complex cellular network, providing a direct mechanism through which immunotherapy can affect bone remodeling [1]. The role of T-cells is complex and depends on their activation state. For instance, non-activated T-cells have been shown to promote osteoclast differentiation, while activated T-cells can suppress osteoclastogenesis through the secretion of cytokines like IFN-γ and IL-4 [26]. In turn, bone cells secrete regulatory molecules that can influence immune cell behavior, reinforcing the reciprocity of this axis [16]. ICIs-induced enhancement of T-cell responses, therefore, has the potential to disrupt bone remodeling by amplifying bone resorption pathways, providing a plausible mechanistic link to bone loss observed in patients undergoing ICIs therapy [9, 20].

The Receptor Activator of Nuclear factor Kappa-B/Receptor Activator of Nuclear factor Kappa-B Ligand/Osteoprogerin (RANK/RANKL/OPG) signaling axis is central to this interaction and represents a major convergence point between skeletal and immune functions [16]. RANKL, expressed by osteoblasts, osteocytes, activated T-cells, B-cells, and bone marrow stromal cells, binds to RANK on osteoclast precursors and mature osteoclasts, thereby promoting their differentiation and resorptive activity [16]. ICIs-driven T-cell activation elevates RANKL expression, thereby stimulating osteoclastogenesis [1, 26]. The clinical relevance of this pathway is underscored by the efficacy of denosumab [38], a monoclonal antibody that neutralizes RANKL and is used to treat bone loss in both oncology and osteoporosis [39]. OPG, a decoy receptor secreted primarily by osteoblasts and stromal cells, binds RANKL and inhibits its interaction with RANK, thereby suppressing osteoclast activity. The RANKL/OPG ratio is a critical determinant of the bone resorption rate [16]. ICIs shift this ratio in favor of resorption by increasing RANKL levels via T-cell activation, establishing a direct link between immune modulation and skeletal degradation [9].

Beyond the RANKL axis, cytokine networks also play a pivotal role in the bone-immune interface. Pro-inflammatory cytokines, such as tumor necrosis factor-alpha (TNF-α), interleukin-1 (IL-1), IL-6, and IL-17, are widely recognized for their pro-resorptive effects [1, 20]. The function of interferon-gamma (IFN-γ) in this process is particularly nuanced. While IFN-γ can directly inhibit osteoclast precursors by interfering with the RANKL signaling cascade, this anti-resorptive effect can be overridden in T-cell rich inflammatory environments [19]. In such conditions of T-cell-mediated inflammation, IFN-γ indirectly stimulates osteoclast formation and promotes bone loss by enhancing T-cell activation and their subsequent secretion of the primary osteoclastogenic factors, RANKL and TNF-α [18].

In addition to bone resorption, the bone formation arm of remodeling is regulated in part by the Wnt signaling pathway. This pathway is crucial for osteoblast proliferation, differentiation, and survival, and its modulation can directly impact bone formation [40]. The canonical Wnt pathway is negatively regulated by proteins such as DKK1 and sclerostin (SOST), which are primarily produced by osteocytes [38]. These inhibitors prevent Wnt ligands from activating their receptors, thereby suppressing osteoblastic activity and bone formation. Although direct evidence linking ICIs to changes in DKK1 or SOST levels remains limited, cytokine alterations induced by ICIs may indirectly influence this pathway. For example, certain proinflammatory cytokines are known to modulate the expression of Wnt inhibitors [16]. Research by Park et al. has suggested that DKK1 also possesses immune-modulatory properties [41] and may be regulated in response to changes in immune signaling cascades. This raises the possibility that ICIs-induced shifts in systemic immune tone could influence Wnt signaling through indirect mechanisms, potentially contributing to an uncoupling of bone formation and resorption [1, 9].

Furthermore, osteocytes are highly sensitive to systemic factors, including hormones and cytokines, which can be altered in the context of endocrine irAEs induced by ICIs [33]. Disruption of endocrine pathways, such as thyroid function or sex hormone levels, may influence osteocyte activity and downstream production of Wnt inhibitors, further compounding skeletal risk. Taken together, the current evidence supports a model in which ICIs, through their primary immune-stimulating action, affect multiple signaling pathways within the BME, ultimately tipping the balance toward bone loss. Understanding these cellular and molecular dynamics is essential for anticipating skeletal complications and for developing targeted strategies to preserve bone health in patients receiving ICIs therapy.

## Systemic Effects of ICIs on Bone Cells and Remodeling

Systemic immune activation triggered by ICIs has both direct and indirect consequences on bone remodeling, potentially disrupting the delicate balance between bone resorption and formation [42]. These effects are mediated by multiple cellular and molecular mechanisms, involving not only osteoclasts and osteoblasts but also osteocytes and their interactions with immune cells, particularly T-lymphocytes.

### Impact on Osteoclastogenesis and Osteoclast Activity

A substantial body of preclinical evidence suggests that ICIs can significantly enhance osteoclast activity and bone resorption.

#### Preclinical Evidence

**Fig. 1:**
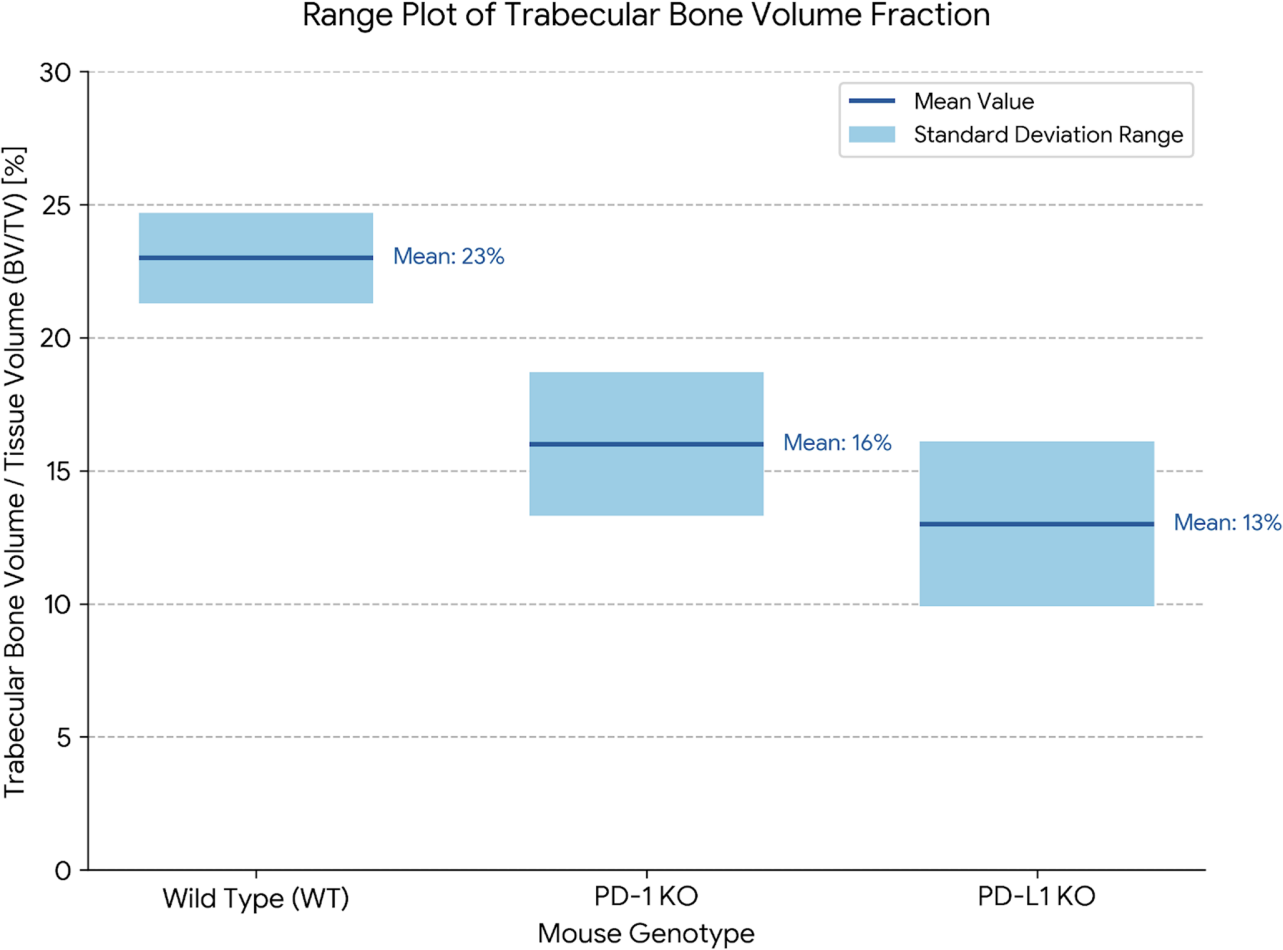
**Trabecular bone volume fraction (BV/TV) in immune checkpoint knockout (KO) mice**. The plot shows the mean (solid line) and standard deviation range (shaded area) for the trabecular bone volume fraction (BV/TV) in the distal femoral metaphysis of 8-week-old male wild type (WT), PD-1 KO, and PD-L1 KO mice. Both KO models exhibit significantly reduced trabecular bone mass compared to WT controls, demonstrating the role of these checkpoint pathways in maintaining basal bone mass. Data presented in this figure were re-plotted from the original study by [22], published in Frontiers, and are used here under the terms of the creative commons attribution license (CC BY).

Studies using genetically modified mice lacking PD-1 (Pdcd1) or PD-L1 (Cd274) revealed altered bone phenotypes, underscoring the role of these checkpoints in skeletal homeostasis [22] (Table 1). At 8 weeks of age, both PD-1 and PD-L1 knockout (KO) mice exhibited significantly reduced trabecular bone volume fraction (BV/TV) compared with wild-type (WT) controls, a key finding conceptually illustrated in Fig. 1. In PD-1 KO mice, this reduction in BV/TV was accompanied by increased trabecular spacing (Tb.Sp), whereas PD-L1 KO mice showed increased Tb.Sp and decreased trabecular thickness (Tb.Th) [22] (Table 1). In PD-L1 KO mice, cortical bone was also compromised, with reduced bone area (B.Ar) and cortical thickness (Ct.Th) and an enlarged marrow area (Ma.Ar) [22] (Table 1). Areal BMD (aBMD) measured by DEXA was significantly lower in PD-L1 KO mice but not significantly changed in PD-1 KO mice at this age; however, PD-1 KO mice experienced a further decline in bone density (measured by µCT) between 8 and 16 weeks [22] (Table 1). Serum analyses indicated elevated RANKL levels in both the KO models. Only PD-L1 KO mice had increased OPG levels, while the RANKL/OPG ratio was significantly increased exclusively in PD-1 KO mice, with no change observed in PD-L1 KO mice [22] (Table 1).

**Table 1:** Summary of key preclinical findings on ICIs effects on bone

Model Type	ICI Target	Sex	Key Bone Parameters Measured	Main Findings	Reference
PD-1 KO Mouse (8 wk)	PD-1	Male	µCT (Trabecular BV/TV, Tb.Sp), Serum (RANKL, OPG, Ratio)	⇓ BV/TV, ⇑ Tb.Sp, ⇑ RANKL, ⇑RANKL/OPG ratio	[22]
PD-L1 KO Mouse (8 wk)	PD-L1	Male	µCT (Trabecular & Cortical), DEXA (aBMD), Serum (RANKL, OPG)	⇓Trabecular & Cortical bone, ⇓ aBMD, ⇑RANKL, ⇑OPG	[22]
PD-1 KO Mouse (16 wk)	PD-1	Male	µCT	⇓Bone density dfurther from 8 wk	[22]
PD-1 KO Mouse (Skeletally Mature)	PD-1	Male & Female	µCT (Trabecular), Biomechanics	Broadly results in bone loss in both sexes; decreased bone strength in adult males and young females.	[25]
Pharmacologic PD-1 Blockade (Mouse, Tumor Model)	PD-1 (Nivolumab)	Male & Female	µCT, Histomorphometry (TRAP), BTMs (CTX-I), Radiography	Inhibited cancer-induced osteoclastogenesis & bone destruction; No effect on normal bone in naive mice.	[24]
Pharmacologic PD-1 Blockade (Mouse, Adjuvant Model)	PD-1 (α-PD-1 Ab)	Female	µCT (Trabecular), Histomorphometry (Osteoclast activity)	Reduced bone mass, increased osteoclast activity; bone loss was T-cell dependent.	[25]
CTLA-4-Ig Admin. (Mouse Arthritis Model)	CTLA-4 (Agonism)	Male	Histology (Osteoclast number, Bone erosion area)	⇓Osteoclast number, ⇓Bone erosion area; No significant change in synovial inflammation.	[21]
In Vitro Osteoclastogenesis	CTLA-4 Protein	N/A	TRAP + Cell Count	Dose-dependent inhibition of RANKL/TNF-induced osteoclastogenesis.	[21]
In Vitro T-cell/Osteoclast Co-culture	T-cell Activation State	N/A	Osteoclastogenesis, Cytokine Expression	Non-activated T-cells: Inc. osteoclastogenesis; Activated T-cells:⇓ osteoclastogenesis.	[26]
In Vitro Osteoclastogenesis (Human PBMCs)	PD-1/PD-L1 Antagonists	N/A	Osteoclastogenesis (TRAP + cell size & number)	Inhibited osteoclastogenesis (e.g., PD-1 Ab, PD-L1 Ab, Nivolumab reduced OC size and/or number).	[43]
Ex Vivo 3D BMU Model	PD-1/PD-L1 Antagonists	N/A	Osteoclast & Osteoblast Markers, Proteomics	⇓Cathepsin K expression (impaired OC differentiation), ⇑ALP activity (enhanced OB activity).	[13]

*Abbreviations: *µCT* micro-computed tomography, *BV*/*TV* bone volume/tissue volume, *aBMD* areal bone mineral density

Taken together, the detailed findings from Greisen et al. provide strong genetic evidence that both PD-1 and PD-L1 are essential for maintaining normal bone homeostasis [22]. This conclusion is strongly supported by other preclinical models, including those using pharmacological blockade, which also demonstrate that the resulting bone loss is critically dependent on T-cells [22, 25]. The key mechanistic insight from this body of preclinical work is the consistent shift in the RANKL/OPG axis towards a pro-resorptive state. Interestingly, Greisen et al. noted that while RANKL was elevated in both knockout models, the RANKL/OPG ratio was only significantly increased in PD-1 KO mice, suggesting there may be subtle mechanistic differences between PD-1 and PD-L1 signaling in bone that require further study. Despite these nuances, the collective preclinical evidence firmly establishes a T-cell-mediated, pro-osteoclastic mechanism for the skeletal effects of PD-1/PD-L1 pathway inhibition, providing a plausible biological basis for the increased fracture risk observed clinically [22].

These findings were confirmed by studies using pharmacological PD-1 blockade therapy. Mice treated with anti-PD-1 antibodies displayed reduced BMD and bone volume fraction, alongside increased osteoclast activity and T-cell expansion within the bone marrow [25]. Notably, this bone loss was absent in T cell-deficient mice, confirming the critical role of T cells in mediating ICIs-induced skeletal effects [25]. However, in a murine bone cancer model, PD-1 inhibition reduced tumor-induced osteoclast formation and bone destruction but did not significantly alter bone structure in non-tumor-bearing animals [24], highlighting the context-dependent nature of these effects.

#### Role of T-Cells

The activation state of T-cells plays a pivotal role in osteoclastogenesis. In vitro T-cell/osteoclast co-culture studies have shown that non-activated T-cells can increase osteoclastogenesis, associated with increased RANKL and TNFα expression, whereas activated T-cells tend to decrease osteoclastogenesis, associated with increased IFNγ and IL-4 expression [26] (Table 1). The critical role of T-cells in mediating skeletal effects following immune checkpoint modulation is further underscored by the findings of [25], where osteoclast-mediated bone loss observed after pharmacological PD-1 blockade was absent in T-cell-deficient mice (Table 1). These findings provide a mechanistic rationale for considering interventions targeting T-cell–osteoclast crosstalk within the bone microenvironment as a strategy to preserve bone integrity.

#### PD-1/PD-L1 Signaling in Osteoclast Lineage Cells

The direct role of PD-1/PD-L1 signaling within osteoclast lineage cells has been explored in various preclinical models, yielding diverse observations [20]. While some studies have suggested that PD-1 deficiency or blockade can reduce osteoclast formation, Wang et al. showed that PD-1 inhibition (nivolumab) inhibited cancer-induced osteoclastogenesis in a tumor model (Table 1) [24]. Other in vitro studies have also indicated a direct inhibitory effect of PD-1/PD-L1 antagonists on osteoclastogenesis [13, 43]. For example, treatment of human PBMCs with PD-1 or PD-L1 antagonists (including nivolumab) inhibits osteoclastogenesis and reduces osteoclast size and/or number [43] (Table 1). Furthermore, in an ex vivo 3D BMU model, PD-1/PD-L1 antagonists decreased Cathepsin K expression, indicating impaired osteoclast differentiation and increased alkaline phosphatase (ALP) activity, suggesting enhanced osteoblast activity [13] (Table 1). These discrepancies highlight the complexity of disentangling direct checkpoint effects on osteoclasts from those mediated indirectly, potentially via T-cell activation or the specific disease context and experimental conditions.

#### CTLA-4 Pathway and Osteoclastogenesis

Evaluating CTLA-4’s role in osteoclast biology is challenging because of the severe phenotype of CTLA-4 knockout mice. However, experimental administration of CTLA-4 protein or CTLA-4-Ig (a CTLA-4 pathway agonist) has been shown to directly affect osteoclastogenesis. In vitro, CTLA-4 protein dose-dependently inhibited RANKL/TNF-induced osteoclastogenesis [21](Table 1). In a mouse arthritis model (hTNFtg mice), administration of CTLA-4-Ig inhibited TNF-induced osteoclast formation and bone erosion [21] (Table 1). Specifically, it decreased the osteoclast number and bone erosion area, with no significant change in synovial inflammation or cartilage degradation. In contrast, administration of CTLA-4-Ig to healthy mice led to increased BMD, bone mass, and bone formation markers, an effect suggested to be mediated by Wnt10b [16] (Table 1). These findings highlight the complex and context-sensitive nature of the influence of the CTLA-4 pathway on bone remodeling. And they, which consistently demonstrate an anti-osteoclastic role for CTLA-4 pathway agonism, therefore suggest that therapeutic blockade of CTLA-4 with an ICIs could remove this natural inhibitory brake, potentially contributing to the increased osteoclast activity observed in some clinical contexts.

#### Sex- and Age-Dependent Effects

The skeletal response to ICIs is significantly modulated by both sex and age. Preclinical evidence strongly supports this, as demonstrated by Greisen et al. , who found that male PD-1 knockout mice show an age-dependent progression of bone loss, with further decreased BMD at 16 weeks compared to 8 weeks [22]. Further highlighting sex-specific vulnerabilities, Joseph et al. reported that global PD-1 deletion results in bone loss in skeletally mature mice of both sexes, but with decreased bone strength observed specifically in adult males and young females [25]. Their work also showed that pharmacological PD-1 blockade in female mice decreased bone mass and increased osteoclast activity (Table 1), confirming that these effects are not limited to one sex. While many preclinical and in vitro studies on ICIs do not explicitly detail sex- or age-specific findings, the observed effects on osteoclastogenesis and bone remodeling, as reported by [13, 43], suggest that these processes, which are known to be influenced by sex hormones and aging, warrant further investigation. Indeed, comprehensive reviews on osteoimmunology emphasize the importance of considering these demographic factors, explaining how natural aging and conditions like postmenopausal osteoporosis exacerbate bone loss [16]. Although foundational studies on osteoclast formation often use models of a single sex [21, 26], their findings contribute to the broader understanding of immune-bone interactions that are likely to be differentially regulated by sex and age.

### Effects on Osteoblast Function and Bone Formation

In contrast to the more extensively studied effects on osteoclasts, the impact of ICIs on osteoblasts and bone formation remains poorly defined, representing a significant gap in the current understanding.

#### Current Knowledge and Gaps

The clinical evidence regarding the impact of ICIs on bone formation markers varies. A recent study by Pantano et al. [1] observed a trend toward reduced serum N-terminal propeptide of type I procollagen (P1NP) levels (a marker of osteoblastic activity) in patients receiving ICIs (Table 2). Elsayed et al. [9] in their review, discuss patterns of BTM changes, including findings such as those from [1], suggesting a potential uncoupling of bone remodeling with some ICIs treatments, analogous to that seen in glucocorticoid-induced osteoporosis [14]. This imbalance between resorption and formation may contribute to changes in BMD and fracture risk in patients treated with ICIs. However, some experimental ex vivo 3D BMU models treated with PD-1/PD-L1 antagonists have reported increased ALP activity, indicative of enhanced osteoblast activity, along with decreased osteoclastogenesis [13] (Table 1). These findings suggest potentially complex, microenvironment-dependent effects. Indeed, the overall impact on bone remodeling is multifaceted, with key preclinical models confirming that the resulting skeletal phenotype is critically dependent on T-cell responses [25].

**Table 2:** Summary of clinical studies on ICIs and bone health (BMD, fractures, ONJ, AFF)

Reference	ICI(s) Studied	Patient Population	Bone Outcome Measured	Key Findings
[13]	PD-1/PD-L1 mono	Advanced solid cancers (no bone mets, no BMAs)	BTMs (CTX, P1NP, OCN)	Transient CTX Dec. (1 mo), then gradual P1NP/OCN Inc. (4–6 mo)
[1]	Anti-PD-1 or Anti-PD-L1 monotherapy	Advanced NSCLC or RCC (no bone mets)	BTMs (CTX-I, P1NP)	After 3 months: CTX-I significantly Inc.; P1NP trend Dec. Inc. CTX-I associated with shorter TTF and OS, correlated with tumor size. 9% developed new lumbar fractures.
[10]	Pembrolizumab, Nivolumab, Ipilimumab (mono/combo)	Melanoma (Stage 3 ICI users vs. Stage 2 non-ICI controls)	Lumbar Spine vBMD, OA parameters	No significant vBMD change in ICI users vs. significant Dec. in controls over 1 year; Effect may depend on baseline vBMD; No effect on OA.
[9, 11]	ICIs not specified	Various Cancers	Major Osteoporotic Fractures (MOF)	> 2-fold increase in MOF rate in year post-ICI vs. year pre-ICI (IRR 2.43)
[11]	Pembrolizumab, Nivolumab, Cemiplimab, Durvalumab, Atezolizumab, Avelumab, Ipilimumab	Melanoma	Major Osteoporotic Fractures (MOF)	Increased MOF risk post-ICI vs. pre-ICI (Year 1 h 1.82, Year 2 h 1.85); Risk factors: prior fracture, age, female, combo ICI.
[12]	Nivolumab, pembrolizumab, atezolizumab, durvalumab, ipilimumab, tremelimumab	Pharmacovigilance study (VigiBase database)	Fracture & Osteoporosis signals (IC))	Osteoporosis was associated with an increased reporting frequency of ICI-related fractures
[34]	Atezolizumab, Avelumab, Cemiplimab, Durvalumab, Nivolumab, Pembrolizumab, Ipilimumab	Pharmacovigilance study (FAERS database, pan-cancer)	Musculoskeletal AEs, Fractures (incl. osteoporotic type)	Fractures are major ICI-induced musculoskeletal AEs. 41.85% of ICI-induced fractures are osteoporotic type. Signals detected for osteoporotic-type fractures with some ICIs
[49]	PD-1, PD-L1, CTLA-4 inhibitors	Various Cancers	Bone AEs, Fractures	Significant RORs: Pathological fracture (3.17), Spinal compression fracture (2.51), Femoral neck fracture (2.38), esp. with PD-1 inhibitors. Vertebral fractures often without prior risk.

### The Role of Osteocytes as Central Regulators

Osteocytes, now recognized as central regulators of skeletal remodeling, are critically positioned to mediate the skeletal effects of ICIs. These embedded mechanosensory cells govern bone homeostasis by producing RANKL and OPG to control osteoclasts, while also secreting sclerostin and DKK1 to inhibit osteoblast function [37]. The systemic immune activation induced by ICIs, characterized by altered cytokine profiles and potential T-cell infiltration into the bone marrow, as suggested by preclinical models of pharmacological PD-1 blockade (see [25] in Table 1), could directly affect osteocyte viability and function. However, this potential link remains a key area of uncertainty. Recognition of the limited data on bone cell biology under ICIs therapy is growing. Several recent reviews and clinical studies highlight the need for longitudinal clinical studies to monitor bone health and for mechanistic in vivo models to understand the underlying cellular pathways [9, 28, 44]. While much of the focus has been on T-cell-osteoclast interactions, the importance of elucidating osteocyte-immune interactions in both metastatic and inflammatory contexts is also emphasized as a critical area for near future research [28].

## Adverse Skeletal Effects of ICIs: Clinical Evidence and Mechanistic Insights

The systemic immunomodulatory effects of ICIs manifest in a range of clinically observable alterations in bone health, spanning from changes in biochemical markers of bone remodeling to an increased incidence of fractures and other musculoskeletal irAEs, as detailed in Table 2.

### Alterations in BTMs and their Clinical Significance

BTMs provide dynamic insights into the activity of bone remodeling and have been used to investigate the skeletal impact of ICIs therapy. However, these findings remain inconsistent across the studies. A prospective exploratory cohort study of nine patients with advanced solid malignancies receiving PD-1/PD-L1 inhibitors (no bone metastases or prior bone-modifying agents (BMA) use) reported complex temporal changes: a transient decrease in the resorption marker C-terminal telopeptide of type I collagen (CTX) in the first month, followed by a gradual increase in the formation markers P1NP and osteocalcin (OCN) after 4–6 months [13] (Table 2). In a different study by [1], after 3 months of anti-PD-1/PD-L1 monotherapy in patients with advanced non-small cell lung cancer (NSCLC) or renal cell carcinoma (RCC) (no bone metastases), levels of CTX-I (a marker of bone resorption) were significantly increased, and this increase was associated with a shorter time to treatment failure (TTF) (*p* = 0.029) and reduced overall survival (OS) (*p* = 0.030), and positively correlated with tumor progression (Table 2). In this cohort, 9% of the patients developed new lumbar fractures within three months of ICIs initiation [1] (Table 2), highlighting the potential for rapid unforeseen skeletal risks.

Multiple reviews, including those by [28] and [9], have documented similar trends observed in some studies, often reporting increased CTX-I and potentially decreased P1NP during ICIs treatment. However, the heterogeneity across studies complicates interpretation. Factors such as cancer type, disease stage, prior treatments, the specific ICIs agent or regimen (monotherapy vs. combination), timing of sampling, and concurrent corticosteroid use, common in the management of irAEs, can all profoundly influence BTM dynamics [45]. These variables contribute to inconsistent outcomes and underscore the individualized nature of ICIs-related skeletal effects. Importantly, the observed association between CTX-I elevation and poor oncologic outcomes [1] (Table 2) suggests that BTMs may reflect broader systemic immune dysregulation or serve as surrogate markers of tumor-immune dynamics. A recent paper on BTM interpretation provides broader context [46] but highlights the need for standardization in studies involving ICIs-treated populations.

### Changes in BMD: Insights from Dual-Energy X-Ray Absorptiometry (DXA) and Quantitative CT (QCT)

**Fig. 2:**
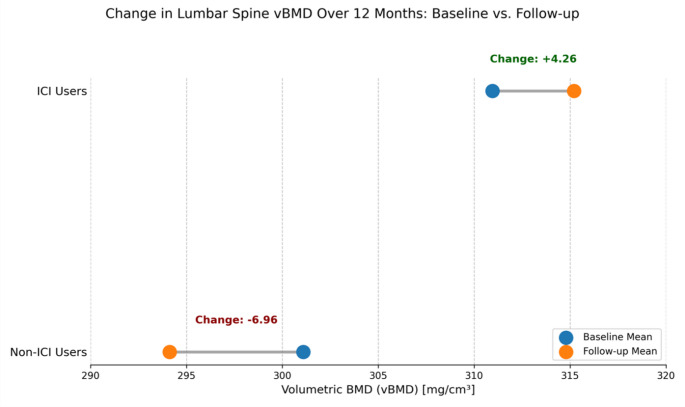
**Change in mean lumbar spine vBMD over 12 months. Baseline vs. follow-up.** The dumbbell plot illustrates the change in mean volumetric bone mineral density (vBMD) from baseline to 12-month follow-up for both ICIs Users and Non-ICIs Users. Data points representing the baseline means and the mean changes (ICIs Users: +4.27 mg/cm³; Non-ICI Users: −6.96 mg/cm³) were extracted directly from Table 3 of the published opportunistic quantitative computed tomography (QCT) study by [10]. This figure visualizes these reported summary values.

Emerging data suggest that ICIs may influence BMD, although findings are currently limited and somewhat paradoxical. Historically, the effect of ICIs on human BMD was unknown [28], but recent studies have begun to shed light. In a machine learning-assisted opportunistic QCT study of melanoma patients, Matheson et al. observed a significant decline in lumbar spine volumetric BMD (vBMD) in non-ICIs users over 12 months, whereas as illustrated in the Dumbbell Plot in (Fig. 2), their findings revealed a significant decline in vBMD for non-ICIs users, while the ICIs-treated cohort remained stable overall. This visual representation of the change from baseline to follow-up highlights the potential protective effect of ICIs therapy The adjusted difference in vBMD change between groups was − 13.04 mg/cm³, favoring stability in the ICIs group [10] (Fig. 2).

However, within the ICIs-treated cohort in that study, patients with lower baseline vBMD were more likely to experience further bone loss, while those with better initial bone health showed stable or improved vBMD [10] (Table 2; Fig. 2). This “*stress test*” hypothesis suggests that ICIs may exacerbate underlying skeletal vulnerability rather than directly cause bone loss in all patients. Such findings point to the importance of baseline risk assessment and individualized monitoring strategies. Prospective, longitudinal studies using standardized BMD assessment methods such as DXA and high-resolution peripheral quantitative computed tomography (HR-pQCT) are essential to fully characterize the effects of ICIs on BMD across cancer types and patient demographics [25, 47].

### Fracture Risk in Patients Receiving ICIs: Evidence from Cohort Studies, Databases, and Meta-Analyses

Fracture risk in ICIs-treated patients is an increasingly recognized concern, conceptually illustrated in Fig. 3. Retrospective analyses and pharmacovigilance databases offer compelling, albeit heterogeneous, evidence. In a large U.S. claims database analysis of melanoma patients, Ye et al. (2024) [11] reported an elevated hazard ratio (HR) for major osteoporotic fractures (MOF) during the first (HR: 1.82; 95% CI: 1.24–2.66) and second years (HR: 1.85; 95% CI: 1.12–2.90) following ICI initiation. Prior fracture, older age, female sex, and combination ICIs therapy were identified as significant risk factors [11] (Table 2).

**Fig 3**

**Forest Plot of Increased MOF Risk Following ICIs Therapy.** The plot synthesizes risk estimates from different cohort studies and analyses. Each horizontal line represents the 95% confidence interval (CI) and the square represents the point estimate of the risk ratio (Hazard Ratio [HR] or Incidence Rate Ratio [IRR]). The vertical dashed line at 1.0 indicates no difference in risk. All estimates fall to the right of this line, consistently demonstrating a statistically significant increase in the risk of MOF after ICIs initiation. Data were extracted from published summary statistics. Specifically, risk estimates for the US cohort (Year 1, Year 2, and Combination Therapy analysis) are sourced directly from the Abstract and Table 3 of [11]. Data for the Canadian cohort are sourced directly from Table 2 of [48], as cited in [9].

**Figure:**
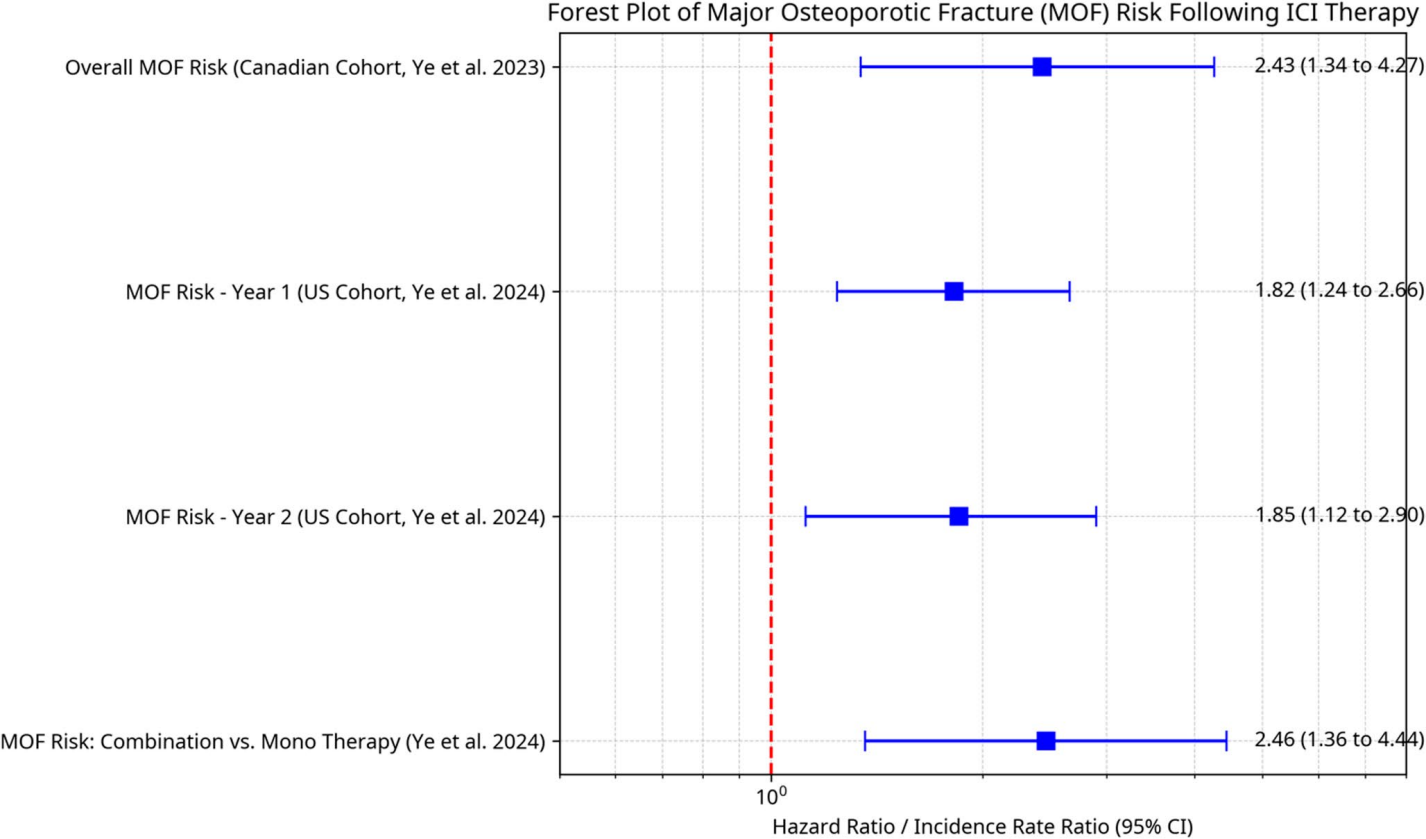

Similarly, a Canadian database study involving various cancer patients found that MOF incidence yielded an incidence rate ratio (IRR) of 2.43 (95% CI: 1.34–4.27) post-ICIs versus pre-ICIs (Table 2) [9, 48]. Pharmacovigilance data from FDA Adverse Event Reporting System (FAERS) and VigiBase further inform these trends. For instance, a study by [49] reported significant reporting odds ratios (ROR)s for pathological, spinal compression, and femoral neck fractures, especially with PD-1 inhibitors (Table 2). Liu et al. [34] also identified fractures as major ICIs-induced musculoskeletal adverse events (AEs), noting that 41.85% of ICIs-induced fractures in FAERS were osteoporotic type, and detected signals for osteoporotic-type fractures with some ICIs (Table 2).

Conversely, a disproportionality analysis by Koseki et al. [12] using VigiBase did not find statistically significant associations between overall fractures or osteoporosis and ICIs (Table 2). However, this study did identify that pre-existing osteoporosis was associated with an increased reporting frequency of ICIs-related fractures (Table 2) [12]. The inconsistency across datasets likely reflects differences in methodology, databases, population characteristics, and background fracture risk.

The disconnection between BMD stability observed in some imaging studies (e.g [10] ,as shown in Fig. 2) (Table 2) and increased fracture rates (illustrated in Fig. 3) highlights a potential role of bone quality deterioration, increased fall risk due to other irAEs (e.g., myositis, neuropathy), or limitations of current imaging modalities in capturing subtle skeletal changes. Further studies are needed to resolve these contradictions and define the fracture mechanisms in ICIs-treated populations.

### Rare Skeletal Events: Osteonecrosis of the Jaw (ONJ) and Atypical Femur Fractures (AFF)

ONJ and AFF are rare skeletal events classically linked to antiresorptive therapies such as bisphosphonates and denosumab [50, 51]. While ICIs are not established causes, there is an emerging concern based on case reports and pharmacovigilance data. ONJ is defined as exposed or probable bone in the maxillofacial region persisting for at least eight weeks, in the absence of jaw radiation or metastasis [52], AFFs are low-trauma femoral shaft fractures with specific radiographic features [53].

Isolated case reports describe ONJ in patients receiving ICIs, including nivolumab, pembrolizumab, and ipilimumab [54, 55]. However, most involve co-administration of antiresorptives or chemotherapy, making causality uncertain. Rare cases of ONJ in BMA-naïve patients receiving ICIs monotherapy have been reported, raising the hypothesis that ICIs-induced immune activation may impair bone healing and remodeling [54]. Pharmacovigilance analyses have not demonstrated strong associations for ONJ with ICIs, and AFFs have not been conclusively associated with ICIs use [55]. Given the rarity of ONJ and AFF, and the polypharmacy common in oncology, it remains difficult to attribute causality to ICIs. Nonetheless, these events warrant vigilance, particularly in patients with additional risk factors or concurrent bone modifying therapies.

## Discussion: Synthesizing Evidence, Addressing Gaps, and Novel Perspectives

### Bridging Preclinical and Clinical Insights into Skeletal Effects of ICIs: The ICIs–Bone Paradox

A notable discrepancy arises when comparing findings from preclinical studies to clinical observations regarding the skeletal effects of ICIs. In mouse models, genetic deletion of PD-1 or PD-L1, as well as pharmacological blockade, consistently produces a catabolic skeletal phenotype characterized by reduced bone mass, compromised trabecular microarchitecture, and increased osteoclast activity mediated by T-lymphocytes [20, 22, 25]. In contrast, one of the few human studies employing opportunistic QCT in melanoma patients found no significant bone loss and even suggested a protective effect of ICIs therapy over one year relative to untreated controls [10]. Simultaneously, multiple large-scale real-world datasets have reported a significantly increased risk of clinical fragility fractures following ICIs initiation [9, 11, 12], highlighting what may be referred to as the “ICIs–bone paradox.”

The presence of bone metastases is a critical confounding factor that may significantly impact the systemic effects of ICIs and their clinical efficacy. Clinically, the data suggest that patients with bone metastases often represent a distinct, more challenging cohort [56, 57]. Large retrospective studies in non-small cell lung cancer have consistently shown that patients with bone metastases have significantly worse progression-free survival (PFS) and overall survival (OS) when treated with ICIs compared to patients without bone involvement [56, 57].

Mechanistically, this diminished efficacy is rooted in the unique, immunosuppressive nature of the bone metastatic microenvironment [28]. Recent groundbreaking research has shown that this is not just a local effect. Bone metastases can drive systemic, extraosseous resistance to ICIs therapy. It has been demonstrated that osseous tumor-conditioned osteoclasts secrete osteopontin (OPN), which enters circulation and impairs the function of essential T-cell populations in distant, non-bone tumors [58]. This finding establishes the skeleton, when burdened with metastases, as an active immunoregulatory organ that can systemically blunt the efficacy of immunotherapy [58]. Therefore, in patients with bone metastases, the powerful local and systemic immunosuppressive signals originating from the metastatic bone niche may overwhelm or alter the direct skeletal effects of ICIs observed in metastasis-free contexts [28].

Several factors may help to reconcile this apparent contradiction. First, skeletal outcomes likely depend on baseline bone health and immune status, as the QCT study noted a more pronounced vBMD decline in ICIs users with an initially low bone density [10]. Second, temporal dynamics may play a key role; while BTMs demonstrate heterogeneous trends, emerging evidence suggests a complex time course involving early resorptive shifts followed by delayed formative responses [1, 13]. Third, the divergent effects of specific ICIs (i.e., PD-1/PD-L1 inhibitors versus CTLA-4 inhibitors) must be considered, given their distinct roles in T-cell modulation and direct interactions at the immune-skeletal interface [21, 35]. Fourth, clinical interpretations are often confounded by patient-specific variables, such as age, sex, cancer type, corticosteroid use, and other irAEs that may increase the risk of falls (e.g., myositis or neuropathy [45]. Specifically, the high-dose corticosteroid regimens frequently required for the management of moderate to severe irAEs are themselves a well-established cause of significant bone loss [7] through glucocorticoid-induced osteoporosis, which uncouples bone remodeling by suppressing formation and promoting resorption [14]. Therefore, this treatment-related toxicity represents a critical confounding factor [45], making it challenging to disentangle the skeletal effects directly attributable to ICIs-mediated immune activation from those caused by the necessary management of its complications [9, 45]. Finally, both preclinical models and clinical imaging approaches have methodological limitations, including species-specific immune responses and the inability of DXA or opportunistic QCT to fully assess the skeletal integrity beyond BMD.

### Bone Quality Beyond BMD: A Critical but Overlooked Component of Skeletal Health

The observation of increased fracture risk in ICIs-treated patients despite apparently stable BMD strongly implicates impaired bone quality as a plausible underlying mechanism. Bone strength is not solely determined by bone mass; it is also influenced by the microarchitecture, matrix composition, and intrinsic material properties [59]. Conventional tools like DXA and QCT quantify density but do not adequately capture these bone qualitative aspects.

The critical determinants of bone quality remain unexamined in the context of ICIs therapy. For instance, the Trabecular Bone Score (TBS), a non-invasive index of trabecular microstructure derived from DXA, has yet to be assessed in ICIs-treated populations [9]. Additionally, parameters such as collagen cross-linking, mineral crystal size and heterogeneity, and the degree of matrix mineralization which collectively influence bone brittleness and resistance to fracture have not been evaluated. Immune activation and cytokine release induced by ICIs may perturb osteoblast or osteocyte function, altering matrix deposition or mineral homeostasis. Such changes could produce a bone that is quantitatively adequate yet structurally fragile. Therefore, addressing this gap is an urgent research priority.

## Conclusion and Future Directions

ICIs have become indispensable in modern oncology and offer unprecedented clinical benefits for a range of malignancies. However, growing evidence indicates that these agents may have unintended consequences on bone health. The pathways targeted by ICIs are not limited to tumor immune evasion but are also involved in physiological bone remodeling through complex osteoimmune interactions [21, 22]. Preclinical studies, particularly those involving PD-1/PD-L1 genetic deletion or blockade, consistently report a catabolic effect on bone, characterized by T-cell–mediated increases in osteoclast activity and subsequent bone loss [22, 25]. However, the clinical data remain contradictory. The only prospective human study using opportunistic QCT found no bone loss or even a potential protective effect in ICIs-treated patients [10], whereas large retrospective cohorts and pharmacovigilance analyses have consistently reported a heightened risk of fragility fractures following ICIs therapy [11, 12]. Data on BTMs are also mixed, with some studies suggesting increased bone resorption and decreased formation [1], while others suggest more dynamic or compensatory changes over time [13]. Although rare skeletal events such as ONJ and AFF appear infrequent, their potential association with ICIs warrants ongoing surveillance [53, 55].

Several significant knowledge gaps hinder a clear understanding of the full impact of ICIs therapy on the skeletal system and limit the development of evidence-based management strategies. To address these uncertainties, near future research must include standardized longitudinal BMD assessment through DXA, particularly of the lumbar spine and proximal femur. Bone turnover should be evaluated using comprehensive panels that measure both resorption (e.g., CTX-I) and formation markers (e.g., P1NP, osteocalcin), as well as key regulators such as RANKL and OPG, captured at multiple time points. The evaluation of bone quality, rather than relying solely on BMD, is also critical. Advanced imaging techniques, such as HR-pQCT and analysis of trabecular microstructure via TBS, may provide valuable insights into architectural deterioration not captured by standard methods [47]. Additionally, there is a need to assess bone material properties more directly. Although challenging in clinical settings, future research should explore feasible approaches to investigate matrix-level changes, including collagen cross-linking, mineral composition, and homogeneity of mineralization. In selected research contexts, this may involve bone biopsy with histomorphometry analysis and mechanical testing.

Future research should aim to identify which patients are most susceptible to ICIs-related skeletal complications by stratifying the risk according to clinical, genetic, and immunological factors. There is a pressing need for the development of reliable biomarkers whether serum-based, imaging-derived, or immunologic that can predict bone turnover changes or fracture risk early in the course of therapy [60]. Mechanistic investigations should focus on elucidating the cellular and molecular pathways that drive skeletal changes during ICIs treatment. This includes understanding how ICIs alter osteocyte function, promote microdamage accumulation, or impair matrix integrity. Preventive strategies to mitigate skeletal risk should be thoroughly tested in future prospective trials. These could include structured exercise programs, nutritional interventions such as calcium and vitamin D supplementation, and the judicious use of bone-modifying agents in selected patients. Moreover, comparative effectiveness research should investigate whether different ICIs classes (i.e., anti-PD-1, anti-PD-L1, and anti-CTLA-4) or combination regimens vary in their skeletal effects. This will further help tailor immunotherapy plans to optimize both oncologic and skeletal outcomes.

The intersection between ICIs and bone biology represents a critical and underexplored dimension of cancer survivorship. While ICIs continue to transform cancer treatment, emerging evidence underscores their potential to disrupt bone integrity through complex immune-mediated mechanisms. Addressing this concern requires an integrated approach combining preclinical studies with prospective, high-resolution clinical investigations. A clearer understanding of these interactions will be essential not only for minimizing skeletal morbidity but also for preserving function and quality of life in the growing population of patients benefiting from immunotherapy.

## Data Availability

No datasets were generated during the current study. Original data approved for reuse by the source publications.
